# Supplementary material for: JAK/STAT Inhibition Normalizes Lipid Composition in 3D Human Epidermal Equivalents Challenged with Th2 Cytokines
Source: Cells. 2024 Apr 29;13(9):760. doi: 10.3390/cells13090760 (PMC11083560; doi:10.3390/cells13090760)
Supplement: Supplementary file 1 [file cells-13-00760-s001.zip › cells-2961365-supplementary.pdf]

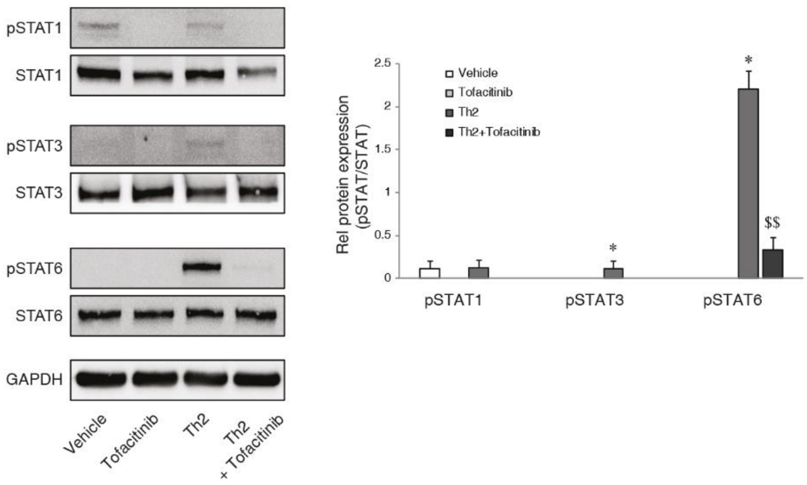

Figure S1. Effects of tofacitinib on STAT signalling (STAT1, STAT3, and STAT6) and their phosphorylated forms. Western blot analysis of pSTAT1/STAT1, pSTAT3/STAT3, and pSTAT6/STAT6 protein expression performed on 3D HEEs and HEEs treated with Th2 cytokines and tofacitinib. Representative blots are shown. GAPDH was used as endogenous loading control. Densitometric scanning of band intensities was performed to quantify the change in protein expression. Data represented the mean ± SD of three independent experiments; *$p<0.05$ vs vehicle; [$$]$p<0.01$ vs Th2.

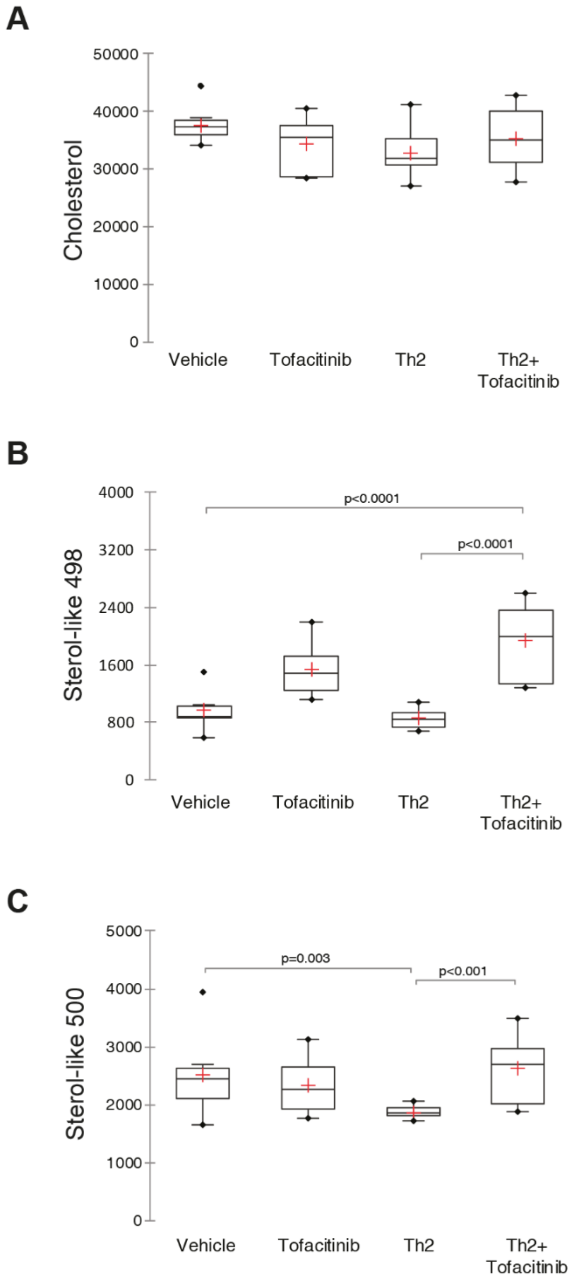

Figure S2. Box plots of cholesterol and sterol like metabolites. Concentration of (**A**) cholesterol and (**B,C**) congeners quantified in lipid extracts of 3D HEEs treated with vehicle, tofacitinib, Th2 cytokines, and with the combined tofacitinib and Th2 cytokines against deuterated cholesterol.

330713 ǀ Deuterated Ceramide LIPIDOMIX®				
**Compound name**	**Formula**	**Molecular weight**	**Exact mass**	**Conc µM**
C16 Ceramide-d7 (d18:1-d7/16:0)	C34H60D7NO3	544.94	544.5560	8
C18 Ceramide-d7 (d18:1-d7/18:0)	C36H64D7NO3	573.00	572.5873	4
C24 Ceramide-d7 (d18:1-d7/24:0)	C42H72D7NO3	657.16	652.6499	8
C24:1 Ceramide-d7 (d18:1-d7/24:1(15Z))	C42H74D7NO3	655.14	654.6656	4
**330731 ǀ EquiSPLASH™ LIPIDOMIX®**				
**Compound name**	**Formula**	**Molecular weight**	**Exact mass**	**Conc µM**
15:0-18:1(d7) PC	C41H73D7NO8P	753.11	752.6061	27
18:1(d7) Lyso PC	C26H45D7NO7P	528.72	528.3921	38
15:0-18:1(d7) PE	C38H67D7NO8P	711.03	710.5591	28
18:1(d7) Lyso PE	C23H39D7NO7P	486.64	486.3451	41
15:0-18:1(d7) PG (Na Salt)	C39H67D7NaO10P	764.02	763.5357	26
15:0-18:1(d7) PI (NH4 Salt)	C42H75D7NO13P	847.13	846.5963	24
15:0-18:1(d7) PS (Na Salt)	C39H66D7NNaO10P	777.02	776.5309	26
15:0-18:1(d7)-15:0 TG	C51H89D7O6	812.37	811.7646	25
15:0-18:1(d7) DG	C36H61D7O5	587.98	587.5506	34
18:1(d7) MG	C21H33D7O4	363.59	363.3366	55
18:1(d7) Chol Ester	C45H71D7O2	658.16	657.6441	30
d18:1-18:1(d9) SM	C41H72D9N2O6P	738.12	737.6397	27
C15 Ceramide-d7 (d18:1-d7/15:0)	C33H58D7NO3	530.92	530.5404	38
**In-house Standards MIX**				
**Compound name**	**Formula**	**Molecular weight**	**Exact mass**	**Conc µM**
Hexadecanoic-d17 Acid (d17-PA)	C16H15D17O2	273.53	273.3469	80
n-Hexadecyl-1,1,2,2-d4 Hexadecanoate-16,16,16-d3 (d17-PA)	C32H57D7O2	487.90	487.5346	40
Glyceryl trihexadecanoate-d98 (d98TG 48:0)	C51D98O6	905.92	905.3515	20
Deuterated cholesterol sulfate sodium salt (d7-CHS)	C27H38D7NaO4S	495.34	495.3376	20
N-palmitoyl-d31-D-erythro-sphingosine (d31-Cer16:0)	C34H37D30NO3	569.10	567.7004	1
Deuterated cholesterol-2,2,3,4,4,6-d6	C27H40D6O	392.70	392.3925	40

Table S1. List of the deuterated internal standards used for extraction protocol and semi-quantitative analyses.

Oligonucleotide sequences (5′-3′)	Amplicone size	Accession number
**ABCA12**<br>sense: ACAGGAATGGCCTTCATCAC<br>antisense: AACATGGTGCCCTGAGAAAC	317 bp	NM_015657.4
**ACADS**<br>sense: CCTCAGCGAACCAGGGAAC<br>antisense: TTCAGAACCCATGAGTCGCC	81 bp	NM_000017.4
**ACAT1**<br>sense: GGAGGCTGGTGCAGGAAA<br>antisense: TCCAATGGGTGTTCTTGTAGC	101 bp	NM_001386677.1
**ACOX1**<br>sense: CGCCGAGAGATCGAGAACAT<br>antisense: GACAGCCACCTCATAACGCT	96 bp	NM_004035.7
**ALOX3**<br>sense: CACCTACACCAATTTCTGCCTTCC<br>antisense: GCCCACGATTTCTGAGACAAAGC	133 bp	NM_021628.3
**ALOX12B**<br>sense: GCTGCTGGTGCTCTGGAC<br>antisense: CTGGCGGATGTCGTGTGAG	151 bp	NM_001139.3
**CA2**<br>sense: AACAATGGTCATGCTTTCAACG<br>antisense: TGTCCATCAAGTGAACCCCAG	128 bp	NM_000067.3
**CASP14**<br>sense: ACATCGCCTACCGACATGATC<br>antisense: CCGGGTCACCTCTGTCAGAA	110 bp	NM_012114.3
**CCL26**<br>sense: GCTGCTTCCAATACAGCCACA<br>antisense:TCCTTGGATGGGTACAGACTTTC	135 bp	NM_001371936.1
**CD36**<br>sense: CTCTTTCCTGCAGCCCAATG<br>antisense: CTGCCACAGCCAGATTGAGA	95 bp	NM_001001548.3
**CERS1**<br>sense: CAATGTGGGCATCCTTGTGCT<br>antisense: AGTAGAGGCGGAACCAGAAC	176 bp	NM_001387443.1
**CERS2**<br>sense: GCCAGGTAGAGCGTTGGTTC<br>antisense: GGCAATGAAGGCAATCAGGT	113 bp	NM_181746.4
**CERS3**<br>sense: TCTCTGCTGACTGCATCTATTG<br>antisense:GAAGCCAGAATCTTTCCAACC	145 bp	NM_001290341.2
**CERS4**<br>sense: GCAGTATCAGCAAGTGTGCG<br>antisense: CCTGTTGCTGATGGACTCGT	124 bp	NM_024552.3
**CERS5**<br>sense: TGACACCCTTTTTGTGATCTTCA<br>antisense: GAAGCATAAGGCCCGATTAT	120 bp	NM_001331069.3

**CERS6**<br>sense: GGGCGGACCTGAAGAACAC<br>antisense: CGCACGGTTTGGCTACAAAT	129 bp	NM_203463.3
**CPT1α**<br>sense: CGTCTTTTGGGATCCACGATT<br>antisense TGTGCTGGATGGTGTCTGTCTC	100 bp	NM_001876.4
**DEGS1**<br>sense: GTCTACACCGACCAGCCG<br>antisense: GCATAGGCCCCAAATATGACC	188 bp	NM_001321541.2
**DEGS2**<br>sense: GGTGCCTGTGCCCTACATTA<br>antisense: GGCCTCAAGCTCCACTCATC	135 bp	NM_206918.3
**DHCR7**<br>sense: GCAGGGGTTGTGAACAAGTAT<br>antisense:GAGACGGCATAGCCAAGGAT	167 bp	NM_001360.3
**DHCR24**<br>sense: AGTCCAGTTCCCCGTTTA<br>antisense: CTTACCCAGCACCTTCAA	163 bp	NM_014762.4
**ELOVL1**<br>sense: CCAAGGTCAAGGCCAACTGA<br>antisense: CTGACGGACACTGCCCTAAG	87 bp	NM_001256401.2
**ELOVL2**<br>sense: CTCCCTCTTGTGCCAGTCAG<br>antisense: GACTCCAGTGCTTGCTCAGA	106 bp	NM_017770.4
**ELOVL3**<br>sense: CAATGAAGCTCCAGGCTCTC<br>antisense: AACCATGCAGGTAAGGCAAC	90 bp	NM_152310.3
**ELOVL4**<br>sense: CCGGAATGGTCAAATCTCTCC<br>antisense: ACACCATCATCATCAAGCCTC	111 bp	NM_022726.4
**ELOVL5**<br>sense: AGGAAGGGTGTAGGGTGAGT<br>antisense: AACCAGTTCCCCATTGCTCA	181 bp	NM_001242830.2
**ELOVL6**<br>sense: CTTTTGTTCAGTTGCCCCGG<br>antisense: TTCGGCTCACCTCTTTTCCC	175 bp	NM_001130721.2
**ELOVL7**<br>sense: TATTCACGGTCACCCACAGC<br>antisense: CCAAACCACCTGCAGCAAAT	199 bp	NM_001104558.2
**FADS-2**<br>sense: TGTCTACAGAAAACCCAAGTGG<br>antisense: TGTGGAAGATGTTAGGCTTGG	128 bp	NM_001281502
**FAS**<br>sense: GACCGCTTCCGAGATTCC<br>antisense: GATGGCAGTCAGGCTCAC	137 bp	NM_004104
**FLG**		

sense: GAAGACAAGGATCGCACCAG<br>antisense: ATGGTGTCCTGACCCTCTTG	76 bp	NM_002016.2
**GAPDH**<br>sense: TGCACCACCAACTGCTTAGC<br>antisense: GGCATGGACTGTGGTCATGAG	198 bp	NM_001289746
**HMGB1**<br>sense: GTGCCTCGCTGAGGAAAAAT<br>antisense: TCCTCCCGACAAGTTTGCAC	105 bp	NM_001363661.2
**HMG-CoA reductase**<br>sense: AGCTACAATGTTGTCAAGAC<br>antisense: GCAGATGGTCAGTGTCAC	97 bp	NM_000859.3
**HMG-CoA synthase**<br>sense: GTATGCCCTGGTAGTTGCAGGAG<br>antisense: TGTTGCATATGTGTCCCACGAA	147 bp	NM_001324220.2
**IL-1α**<br>sense: CGCCAATGACTCAGAGGAAGA<br>antisense: AGGGCGTCATTCAGGATGAA	120 bp	NM_000575
**IL-1β**<br>sense: CTGAGCTCGCCAGTGAAATG<br>antisense: TTTAGGGCCATCAGCTTCAAA	77 bp	NM_000576.2
**IL-6**<br>sense: AGCCACTCACCTCTTCAGAACG<br>antisense: GGTTCAGGTTGTTTTCTGCCAG	141 bp	NM_000600
**IL-8**<br>sense: CTTGGCAGCCTTCCTGATTTC<br>antisense: TTCTGTGTTGGCGCAGTGTG	168 bp	NM_000584
**IVL**<br>sense: ACCCATCAGGAGCAAATGAAA<br>antisense: GCTCGACAGGCACCTTCTGGC	67 bp	NM_005547.4
**K6**<br>sense: AGTCCTGCTTCTCTTC<br>antisense: CTGCTGTGGCTCCTGATG	107 bp	NM_005554.4
**K10**<br>sense: TGATGTGAATGTGGAAATGAATGC<br>antisense: GTAGTCAGTTCCTTGCTCTTTTCA	147 bp	NM_000421.5
**LOR**<br>sense: TCATGATGCTACCCGAGGTTTG<br>antisense: CAGAACTAGATGCAGCCGGAGA	87 bp	NM_000427.3
**PPARγ**<br>sense: GCCAAGCTGCTCCAGAAAAT<br>antisense: TGATCACCTGCAGTAGCTGCA	73 bp	NM_138711
**PDPN**<br>sense: TGACTCCAGGAACCAGCGAAG<br>antisense: GCGAATGCCTGTTACACTGTTGA	86 bp	NM_006474.5
**PLIN1**<br>sense: ACATTAAAGGGAAGAAGTTGAAGC	93 bp	NM_002666.5

antisense: TTCTCCTGCTCAGGGAGGT		
**PLIN2**<br>sense: TGAGATGGCAGAGAACGGTG<br>antisense: GCAATTTGCGGCTCTAGCTT	90 bp	NM_001122.4
**PLIN5**<br>sense: AAGGCCCTGAAGTGGGTTC<br>antisense: GCATGTGGTCTATCAGCTCCA	192 bp	NM_001013706.3
**SCD-1**<br>sense: CATAATTCCCGACGTGGCTTT<br>antisense:AGGTTTGTAGTACCTCCTCTGGAACA	150 bp	NM_005063
**SLC27A4 (FATP4)**<br>sense: AAATCGGGGAGTTCTACGGC<br>antisense: CAGGATGCGGCTGTTGAAAC	95 bp	NM_198580.3
**SPT**<br>sense: TGGTCATTTGGCCCAGGTC<br>antisense: TTCCAACCATTGGC TTCACATC	121 bp	NM_004863.4
**SREBP-1**<br>sense: GGAGCCATGGATTGCACTTT<br>antisense: TCAAATAGGCCAGGGAAGTCA	77 bp	NM_001005291
**SREBP-2**<br>sense: CCGCCTGTTCCGATGTACAC<br>antisense: TGCACATTCAGCCAGGTTCA	56 bp	NM_004599.4
**TGM1**<br>sense: TCTTCAAGAACCCCCTTCCC<br>antisense: TCTGTAACCCAGAGCCT	69 bp	NM_000359.3
**TSLP**<br>sense: TGGGGGCTGGTGCCCTACTC<br>antisense: AATTGGCCCCGAAGGCTGGC	118 bp	NM_033035.5

Table S2. Forward (F) and reverse (R) primers used for the Real time RT-PCR analysis.

**Differentiation**			
	**Tofacitinib**	**Th2**	**Th2+Tofacitinib**
CASP14	0,91±0,07	0,52±0,24	0,85±0,13
FLG	1,35±0,69	0,55±0,26	0,96±0,28
K6	1,41±0,55	1,93±0,22	1,20±0,28
K10	1,47± 0,65	1,16±0,52	1,15±0,24
LOR	1,00±0,41	0,58±0,28	0,86±0,36
IVL	1,03±0,42	1,00±0,04	0,96±0,18
TGM1	1,29±1,05	1,10±0,52	1,28±1,06
**Inflammation**			
CCL26	1,37±0,95	7,47±3,07	0,87±0,47
IL-1α	0,95±0,17	1,89±0,12	0,79±0,04
IL-1β	0,93±0,18	1,72±0,23	0,78±0,24
IL-6	0,55±0,22	8,20±1,83	0,62±0,19
IL-8	1,09±0,22	1,90±0,73	0,98±0,31
PDPN	0,81±0,17	2,49±0,32	0,83±0,11
TSLP	1,18±0,21	0,93±0,20	1,15±0,28
**Lipid metabolism**			
ABCA12	1,17±0,46	1,03±0,16	1,01±0,31
ALOX3	0,79±0,17	1,26±0,44	0,89±0,29
ALOX12B	0,80±0,23	1,41±0,35	1,16±0,85
**CA2**	0,85±0,005	5,03±1,55	0,93±0,30
CD36	1,84±1,13	1,40±0,72	1,20±0,07
CERS1	0,58±0,36	1,07±0,33	1,09±0,30
CERS2	1,21±0,60	1,04±0,22	1,08±0,42
CERS3	0,96±0,23	0,73±0,19	0,92±0,26
CERS4	0,88±0,13	0,73±0,17	0,72±0,17
CERS5	1,06±0,42	1,13±0,50	1,11±0,51
CERS6	0,90±0,23	1,10±0,35	0,93±0,31
DEGS1	0,92±0,19	1,03±0,34	0,91±0,15
**DEGS2**	1,16±0,39	1,59±0,29	0,70±0,16
DHCR7	1,35±0,58	0,95±0,08	1,19±0,21
DHCR24	1,48±0,87	1,03±0,23	1,13±0,34
**ELOVL1**	1,24±0,61	0,63±0,12	0,95±0,47
ELOVL2	1,31±0,66	1,25±1,27	0,78±0,17
**ELOVL3**	0,72±0,30	0,21±0,16	0,71±0,27
**ELOVL4**	1,06±0,11	0,47±0,17	0,86±0,11
ELOVL5	0,69±0,33	1,41±0,59	0,97±0,19
ELOVL6	1,10±0,13	1,02±0,26	0,93±0,27
ELOVL7	1,56± 0,80	1,29±0,58	0,89±0,09
FADS	1,51±1,33	0,77±0,45	1,46±1,42
FAS	1,23±0,57	1,04±0,47	0,94±0,07
HMGB1	0,93±0,15	1,02±0,17	1,07±0,26

HMG-CoA reductase	1,12±0,52	1,39±0,99	0,93±0,13
HMG-CoA synthase	1,19±0,38	0,94±0,28	1,01±0,39
PLIN1	0,81±0,29	0,40±0,11	0,96±0,20
PLIN2	1,19±0,34	0,21±0,11	0,73±0,03
PLIN5	1,53±1,71	0,89±0,38	1,05±1,09
SCD	1,19±0,03	0,85±0,06	0,98±0,17
SLC27A4	1,26 ±0,57	0,89±0,37	0,91±0,06
SPT	1,14±0,19	0,55±0,05	1,08±0,19
Mitochondrial function			
ACADS	*1,08*±0,18	1,31±*0,23*	*1,03*±0,25
**ACAT1**	*0,90*±0,17	*1,62*±0,16	*1,09*±0,24
ACOX1	*1,02*±0,26	*1,35*±0,16	*0,93*±0,11
CPT1α	*0,90*±0,15	*1,53*±0,14	*1,10*±0,14
**Transcription Factors**			
SREBP1	1,78±1,15	0,89±0,54	1,05±0,19
SREBP2	1,37±0,42	0,89±0,51	0,94±0,12
PPARγ	1,09±0,58	0,57±0,27	0,97±0,40

Table S3. mRNA expression of inflammatory and lipid genes in treated HEEs compared to vehicle. Mean values and standard deviation (SD) of fold changes (FC) of mRNA expression of inflammatory and lipid genes in Th2 and tofacitinib-treated HEEs compared to vehicle. The genes whose transcription was significantly modified with treatments are reported in bold.

Compound	Technique	Neutral Mass	p ([Th2] vs [Th2+T] vs [T] vs [V])	q-value	p ([Th2+T] vs [Th2])	p ([T] vs [Th2])	p ([V] vs [Th2])	p ([V] vs [Th2+T])	p ([V] vs [T])
**FA 16:1n-7**	GCMS (TMS)	311.3000	0.0000	0.000	0.000	0.000	0.000		
**FA 27:0**	LCMS (HR)	410.4122	0.0075	0.030			0.008		
**FA 29:0**	LCMS (HR)	438.4433	0.0074	0.030			0.023	0.030	
**FA 30:0**	LCMS (HR)	452.4591	0.0079	0.030			0.005		
**Sterol-like 458**	GCMS (TMS)	458.0000	0.0002	0.002	0.007	0.000			0.004
**Sterol-like 498**	GCMS (TMS)	498.0000	0.0000	0.000	0.000	0.000		0.000	0.001
**HexCer 42:1;O2**	LCMS (HR)	811.6972	0.0143	0.047			0.049	0.030	0.026
**HexCer 44:1;O2**	LCMS (HR)	839.7276	0.0075	0.030			0.049	0.025	0.009
**HexCer 48:2;O2**	LCMS (HR)	893.7699	0.0036	0.021	0.016	0.029	0.004		
**Cer[NH]40:1**	LCMS (HR)	637.5973	0.0140	0.047		0.048			
**TG 44:2**	LCMS (HR)	746.6431	0.0003	0.003	0.001	0.001		0.034	0.046
**TG 46:3**	LCMS (HR)	772.6581	0.0000	0.000	0.000	0.000		0.006	0.020
**TG 46:2**	LCMS (HR)	774.6750	0.0000	0.000	0.000	0.000	0.017	0.000	0.000
**TG 46:1**	LCMS (HR)	776.6905	0.0001	0.001	0.000	0.001		0.027	
**TG 48:4**	LCMS (HR)	798.6726	0.0004	0.004	0.001	0.003			
**TG 48:3**	LCMS (HR)	800.6894	0.0000	0.000	0.000	0.000	0.000	0.000	0.000
**TG 48:2**	LCMS (HR)	802.7060	0.0000	0.000	0.000	0.000	0.024	0.003	0.018
**TG 48:1**	LCMS (HR)	804.7221	0.0070	0.030	0.007	0.021			
**TG 48:0**	LCMS (HR)	806.7366	0.0020	0.013			0.001		0.045
**TG 50:5**	LCMS (HR)	824.6894	0.0079	0.030	0.008	0.036			
**TG 50:4**	LCMS (HR)	826.7046	0.0000	0.000	0.000	0.000	0.009	0.000	0.001
**TG 50:3**	LCMS (HR)	828.7207	0.0000	0.000	0.000	0.000	0.002	0.000	0.004
**TG 50:2**	LCMS (HR)	830.7370	0.0008	0.006	0.001	0.004			
**TG 51:2**	LCMS (HR)	844.7524	0.0010	0.008	0.001	0.018	0.014		
**TG 52:5**	LCMS (HR)	852.7207	0.0000	0.000	0.000	0.001		0.002	0.035
**TG 52:4**	LCMS (HR)	854.7361	0.0000	0.000	0.000	0.000		0.000	0.003
**TG 52:3**	LCMS (HR)	856.7523	0.0001	0.001	0.000	0.001		0.016	
**TG 53:3**	LCMS (HR)	870.7668	0.0010	0.008	0.001	0.006	0.030		
**TG 54:4**	LCMS (HR)	882.7672	0.0006	0.005	0.001	0.014		0.007	
**DG 34:0**	LCMS (HR)	596.5380	0.0076	0.030			0.016	0.014	0.037
**DG 34:2**	LCMS (HR)	592.5067	0.0000	0.000	0.000	0.001	0.001		
**DG 35:0**	LCMS (HR)	610.5536	0.0065	0.030			0.010	0.014	0.046
**DG 36:1**	LCMS (HR)	622.5536	0.0000	0.000	0.000	0.000		0.004	0.000
**DG 37:0**	LCMS (HR)	638.5849	0.0076	0.030			0.015	0.011	
**DG 38:0**	LCMS (HR)	652.6006	0.0055	0.029			0.007	0.015	0.049
**DG 38:1**	LCMS (HR)	650.5849	0.0012	0.009	0.039	0.002			0.013
**DG 38:2**	LCMS (HR)	648.5693	0.0144	0.047	0.039	0.027			
**DG 40:0**	LCMS (HR)	680.6319	0.0105	0.038			0.013	0.027	
**PC 32:2**	LCMS (HR)	729.5315	0.0025	0.015	0.003	0.011			
**PC 34:5**	LCMS (HR)	751.5141	0.0024	0.015	0.003	0.017			
**PC 36:2**	LCMS (HR)	785.5950	0.0095	0.035		0.020	0.013		
**PC 36:1**	LCMS (HR)	787.6070	0.0106	0.038		0.009			
**PC 38:2**	LCMS (HR)	813.6253	0.0056	0.029	0.049	0.008	0.013		
**PC 40:4**	LCMS (HR)	837.6184	0.0063	0.030		0.019	0.007		
**LPC 20:4**	LCMS (HR)	543.3303	0.0022	0.014		0.002			0.019
**SM 40:2;O2**	LCMS (HR)	784.6493	0.0053	0.029				0.007	0.013
**SM 44:1;O2**	LCMS (HR)	842.7179	0.0066	0.030	0.030	0.012			
**PE 32:2**	LCMS (HR)	687.4836	0.0000	0.000	0.000	0.000	0.000		
**PE 32:1**	LCMS (HR)	689.4989	0.0124	0.043	0.037		0.016		
**PE 34:3**	LCMS (HR)	713.4995	0.0020	0.013	0.002	0.012	0.024		
**PE O-38:3**	LCMS (HR)	755.5821	0.0061	0.030			0.013	0.010	0.048

Compound	FC (abs) ([Th2] vs [V])	Regulation	FC (abs) ([Th2+T] vs [V])	Regulation	FC (abs) ([T] vs [V])	Regulation
**FA 16:1n-7**	2.098	down	1.045	up	1.034	up
**FA 27:0**	2.229	down	1.839	down	1.316	down
**FA 29:0**	2.126	down	2.061	down	1.171	down
**FA 30:0**	1.953	down	1.565	down	1.422	down
**Sterol-like 458**	1.089	up	1.183	down	1.311	down
**Sterol-like 498**	1.120	down	1.919	up	1.546	up
**HexCer 42:1;O2**	1.505	down	1.558	down	1.574	down
**HexCer 44:1;O2**	1.657	down	1.755	down	1.920	down
**HexCer 48:2;O2**	1.964	down	1.113	down	1.166	down
**Cer[NH]40:1**	1.004	down	1.381	up	1.466	up
**TG 44:2**	1.246	down	1.488	up	1.461	up
**TG 46:3**	1.427	down	1.689	up	1.569	up
**TG 46:2**	1.337	down	1.784	up	1.650	up
**TG 46:1**	1.301	down	1.410	up	1.328	up
**TG 48:4**	1.367	down	1.507	up	1.365	up
**TG 48:3**	1.743	down	1.985	up	1.815	up
**TG 48:2**	1.507	down	1.712	up	1.534	up
**TG 48:1**	1.387	down	1.254	up	1.169	up
**TG 48:0**	1.419	down	1.169	down	1.246	down
**TG 50:5**	1.359	down	1.477	up	1.291	up
**TG 50:4**	1.428	down	1.817	up	1.635	up
**TG 50:3**	1.720	down	1.933	up	1.672	up
**TG 50:2**	1.463	down	1.430	up	1.292	up
**TG 51:2**	1.548	down	1.190	up	1.016	down
**TG 52:5**	1.283	down	1.788	up	1.491	up
**TG 52:4**	1.265	down	1.789	up	1.542	up
**TG 52:3**	1.442	down	1.674	up	1.443	up
**TG 53:3**	1.396	down	1.186	up	1.083	up
**TG 54:4**	1.090	down	1.538	up	1.356	up
**DG 34:0**	2.502	down	2.553	down	2.253	down
**DG 34:2**	1.225	down	1.095	up	1.002	up
**DG 35:0**	2.765	down	2.663	down	2.265	down
**DG 36:1**	1.174	up	1.304	down	1.424	down
**DG 37:0**	2.623	down	2.727	down	2.157	down
**DG 38:0**	3.331	down	2.982	down	2.495	down
**DG 38:1**	1.085	up	1.180	down	1.338	down
**DG 38:2**	1.091	up	1.172	down	1.188	down
**DG 40:0**	3.046	down	2.729	down	2.335	down
**PC 32:2**	1.430	down	1.382	up	1.247	up
**PC 34:5**	1.343	down	1.681	up	1.440	up
**PC 36:2**	1.558	up	1.121	up	1.026	up
**PC 36:1**	1.422	up	1.006	down	1.132	down
**PC 38:2**	1.537	up	1.081	up	1.025	down
**PC 40:4**	1.376	up	1.171	up	1.038	up
**LPC 20:4**	1.143	up	1.271	down	1.587	down
**SM 40:2;O2**	1.246	up	1.438	up	1.398	up
**SM 44:1;O2**	1.094	up	1.193	down	1.240	down
**PE 32:2**	2.467	down	1.054	up	1.023	up
**PE 32:1**	1.512	down	1.051	down	1.077	down
**PE 34:3**	1.497	down	1.156	up	1.041	up
**PE O-38:3**	1.490	up	1.514	up	1.388	up

Table S4. Abundance profiles of FAs, Sterol-like, HexCers, TGs, DGs, PCs, SMs, Pes in HEEs treated with tofacitinib and Th2 cytokines and their combination. q-values, p-values and fold changes (FC) of 51 significative lipid species obtained after one-way ANOVA (V= vehicle; T= tofacitinib).

Variable	V	T	Th2	Th2+T	SD V	SD T	SD Th2	SD Th2+T	FC Th2/V	FC Th2+T/ Th2	Kruskal-Wallis
**HexCer 34:1;O2**	79.013	51.642	44.198	50.746	52.933	16.660	13.460	14.488	0.559	0.983	0.497
**HexCer 36:1;O2**	33.712	14.759	16.055	12.733	51.408	15.075	17.516	12.974	0.476	0.863	0.668
**HexCer 38:1;O2**	17.115	9.819	11.283	11.644	8.734	4.457	3.687	5.754	0.659	1.186	0.267
**HexCer 40:1;O2**	35.077	21.850	28.692	26.606	6.056	7.151	6.028	4.312	0.818	1.218	**0.025**
**HexCer 42:1;O2**	93.709	59.554	61.659	60.280	20.258	11.331	8.097	12.543	0.658	1.012	**0.005**
**HexCer 44:1;O2**	55.407	26.880	32.056	29.213	32.243	10.529	14.840	10.094	0.579	1.087	0.078
**HexCer 46:1;O2**	12.064	5.915	7.430	6.153	3.889	2.399	1.431	2.612	0.616	1.040	**0.012**
**HexCer 40:2;O2**	4.355	3.736	2.633	3.863	1.752	1.476	0.723	1.355	0.605	1.034	0.296
**HexCer 42:2;O2**	19.130	13.290	9.507	12.868	9.409	3.526	3.106	5.081	0.497	0.968	0.176
**HexCer 44:2;O2**	17.158	10.490	7.054	10.651	9.076	5.404	3.766	4.684	0.411	1.015	0.113
**HexCer 46:2;O2**	10.694	6.295	4.162	5.739	7.360	5.088	3.329	4.162	0.389	0.912	0.174
**HexCer 46:1;O2**	12.064	5.915	7.430	6.153	3.889	2.399	1.431	2.612	0.616	1.040	**0.012**

Table S5. Abundance profile of HexCers in HEEs treated with tofacitinib and Th2 cytokines and their combination. Mean values and standard deviation (SD) of amounts (pmol/mg protein) of HexCers and p-valued obtained after Kruskal-Wallis test (V= vehicle; T= tofacitinib).

Variable	V	T	Th2	Th2+T	SD V	SD T	SD Th2	SD Th2+T	FC Th2/V	FC Th2+T/ Th2	Kruskal-Wallis
**12Me-C15:0**	134.120	87.798	80.242	97.606	50.099	21.189	26.972	48.949	0.598	1.216	0.071
**13Me-C15:0**	99.972	68.128	63.521	79.203	33.887	14.897	20.403	38.144	0.635	1.247	0.079
**14Me-C17:0**	604.961	399.191	379.805	440.240	218.028	47.375	115.049	181.997	0.628	1.159	**0.012**
**15Me-C17:0**	347.713	240.652	229.746	278.781	104.273	37.014	59.023	116.545	0.661	1.213	0.051
**FA 12:0**	746.474	425.844	251.069	345.680	669.040	314.172	94.576	130.755	0.336	1.377	0.060
**FA 13:0**	61.920	39.019	29.763	44.321	28.350	15.302	9.395	28.880	0.481	1.489	**0.015**
**FA 14:0**	4614.256	3249.785	2699.352	3571.032	1407.297	461.435	559.758	1393.404	0.585	1.323	**0.002**
**FA 14:1**	24.748	19.677	13.803	22.746	14.323	7.632	7.404	15.046	0.558	1.648	0.195
**FA 15:0**	771.658	516.667	459.158	590.885	248.618	75.206	85.815	211.943	0.595	1.287	**0.004**
**FA 16:0**	57609.383	39020.630	37581.088	42919.251	28832.940	6709.082	7206.616	14368.278	0.652	1.142	0.060
**FA 16:1n-10**	82.528	80.832	93.287	86.226	11.630	13.787	13.153	15.123	1.130	0.924	0.186
**FA 16:1n-7**	1324.638	1421.656	607.980	1473.994	466.981	488.951	121.223	658.808	0.459	2.424	**<0.0001**
**FA 17:0**	2042.903	1390.867	1354.314	1599.834	668.251	195.520	307.664	603.101	0.663	1.181	0.053
**FA 17:1**	7073.464	4698.568	5634.488	5714.523	4176.796	2732.069	4477.394	4651.240	0.797	1.014	0.535
**FA 18:0**	78785.285	51377.971	50595.111	55167.599	46859.494	10374.799	10109.213	17694.526	0.642	1.090	0.148
**FA 18:1n-9**	5048.400	4482.580	4372.361	4791.400	1405.642	1406.185	1567.190	2041.068	0.866	1.096	0.782
**FA 18:1 (II)**	4315.466	4016.429	4312.789	4383.628	1991.115	1960.945	2614.538	2770.871	0.999	1.016	0.972
**FA 18:2**	112.784	100.204	94.456	111.899	21.380	21.722	9.356	18.749	0.837	1.185	**0.049**
**FA 19:0**	156.885	104.965	112.427	124.687	56.707	20.483	30.721	51.201	0.717	1.109	0.107
**FA 20:0**	913.234	675.365	670.214	774.540	224.648	107.328	94.006	233.001	0.734	1.156	**0.032**
**FA 20:1n-9**	95.120	99.206	110.367	115.001	32.011	23.046	20.420	36.468	1.160	1.042	0.583
**FA 20:1 (II)**	214.719	214.669	201.909	240.973	129.009	108.682	110.769	151.318	0.940	1.193	0.936
**FA 22:0**	190.982	156.318	144.019	179.501	58.765	36.965	36.526	55.562	0.754	1.246	0.190
**FA 24:0**	335.140	331.402	313.659	356.055	144.769	68.861	66.704	80.639	0.936	1.135	0.591
**FA 24:1**	29.063	31.944	31.600	33.897	20.159	16.277	20.905	15.798	1.087	1.073	0.889
**FA 26:0**	156.473	158.371	144.473	175.473	94.857	68.117	50.555	53.186	0.923	1.215	0.705
**FA 27:0**	164.838	120.719	72.652	90.891	52.881	26.180	21.196	36.599	0.441	1.251	**<0.0001**
**FA 28:0**	808.332	639.140	562.844	604.151	335.814	281.797	304.665	297.498	0.696	1.073	0.121
**FA 29:0**	49.100	39.965	20.905	21.434	24.326	18.487	6.059	5.697	0.426	1.025	**0.000**
**FA 30:0**	143.497	101.409	76.031	101.650	27.063	22.282	24.535	55.565	0.530	1.337	**0.000**
**FOH 16:0**	64.286	42.821	39.126	42.448	24.773	11.720	14.048	14.838	0.609	1.085	0.052
**FOH 18:0**	723.319	523.187	539.635	413.343	273.845	77.802	134.476	54.287	0.746	0.766	**0.000**
**FOH 17:1**	128.305	123.237	74.699	124.253	41.077	43.838	14.942	69.996	0.582	1.663	**0.015**
**Cholesterol**	37497.070	34373.354	32843.825	35193.946	2615.163	4651.090	4214.859	5189.979	0.876	1.072	0.089
**Sterol-like 458**	856.041	682.010	924.804	750.019	231.427	187.419	121.773	169.203	1.080	0.811	**0.012**
**Sterol-like 486**	22.627	19.929	22.829	23.364	11.482	8.011	5.705	9.302	1.009	1.023	0.683
**Sterol-like 498**	965.622	1535.763	855.508	1933.638	267.166	362.499	137.342	509.651	0.886	2.260	**<0.0001**
**Sterol-like 500**	2521.194	2332.211	1869.067	2623.533	696.018	461.251	96.054	565.419	0.741	1.404	**0.002**
**Sterol-like 520**	1719.558	1872.025	703.946	2299.893	1362.577	1517.390	762.844	2173.004	0.409	3.267	0.072

Table S6. Abundance profile of FFAs, FOHs, cholesterol, and related metabolites in HEEs treated with tofacitinib and Th2 cytokines and their combination. Mean values and standard deviation (SD) of amounts (pmol/mg protein) of FFAs, FOHs, cholesterol, and related metabolites and p-valued obtained after Kruskal-Wallis test (V= vehicle; T= tofacitinib).

Variable	V	T	Th2	Th2+T	SD V	SD T	SD Th2	SD Th2+T	FC Th2/V	FC Th2+T/ Th2	Kruskal-Wallis
**DG 28:0**	19.683	10.759	17.689	14.424	12.026	5.390	11.821	9.271	0.899	0.815	0.451
**DG 29:0**	23.762	25.662	18.568	16.748	9.798	25.075	12.616	13.075	0.781	0.902	0.695
**DG 30:0**	46.069	36.022	42.880	35.864	19.041	11.605	18.774	16.274	0.931	0.836	0.685
**DG 30:1**	38.240	36.252	29.614	37.579	6.956	6.656	6.104	3.481	0.774	1.269	0.114
**DG 31:0**	42.771	25.468	30.272	24.532	30.330	7.384	9.166	5.251	0.708	0.810	0.480
**DG 32:0**	333.420	181.093	197.919	171.760	135.292	31.072	59.545	44.963	0.594	0.868	**0.023**
**DG 32:1**	135.598	123.472	108.219	119.027	32.500	13.641	6.248	14.216	0.798	1.100	**0.046**
**DG 32:2**	120.121	85.260	69.163	84.411	51.033	7.605	17.491	16.011	0.576	1.220	**0.047**
**DG 33:0**	35.990	17.165	18.095	16.055	16.333	6.428	4.952	3.339	0.503	0.887	**0.012**
**DG 33:1**	7.155	3.915	4.339	4.715	4.350	1.229	1.550	1.213	0.606	1.087	0.397
**DG 34:0**	5134.489	2102.854	1881.575	1810.608	2990.608	629.909	504.398	346.880	0.366	0.962	**0.006**
**DG 34:1**	244.192	186.523	221.503	199.851	57.286	23.834	39.157	37.926	0.907	0.902	0.191
**DG 34:2**	268.442	270.079	218.899	293.857	21.410	33.969	13.325	23.741	0.815	1.342	**0.003**
**DG 34:3**	16.132	12.120	14.102	13.384	5.865	5.082	6.448	4.271	0.874	0.949	0.718
**DG 35:0**	110.479	44.691	36.214	36.949	67.523	13.943	9.703	6.878	0.328	1.020	**0.004**
**DG 36:0**	12903.171	4684.880	5277.373	5095.372	7720.221	2721.892	1505.703	1097.572	0.409	0.966	**0.014**
**DG 36:1**	192.721	135.049	226.648	147.441	16.564	7.085	23.803	6.563	1.176	0.651	**0.000**
**DG 36:2**	248.603	230.415	276.973	229.306	35.664	44.886	40.916	25.512	1.114	0.828	0.183
**DG 36:3**	35.810	33.380	34.033	32.766	12.245	10.140	4.509	3.563	0.950	0.963	0.950
**DG 37:0**	32.607	13.647	11.495	10.630	20.477	3.939	4.306	2.087	0.353	0.925	**0.004**
**DG 38:0**	213.974	75.161	54.717	60.469	161.387	28.100	13.727	12.436	0.256	1.105	**0.004**
**DG 38:1**	28.342	21.253	31.180	24.289	1.168	1.994	5.941	4.200	1.100	0.779	**0.007**
**DG 38:2**	48.003	40.961	52.933	41.288	5.014	8.388	10.332	7.141	1.103	0.780	0.122
**DG 39:0**	17.907	8.552	8.423	8.094	10.540	3.030	1.971	2.531	0.470	0.961	**0.031**
**DG 40:0**	94.742	35.370	26.530	29.253	69.975	11.874	6.213	4.936	0.280	1.103	**0.004**
**DG 40:1**	5.090	4.772	3.014	3.393	0.936	3.364	0.712	0.985	0.592	1.126	**0.039**

Table S7. Abundance profile of the detected DGs in HEEs treated with tofacitinib and Th2 cytokines and their combination. Mean values and standard deviation (SD) of DGs amounts (pmol/mg protein) and p-values obtained after Kruskal-Wallis test (V= vehicle; T= tofacitinib).

Variable	V	T	Th2	Th2+T	SD V	SD T	SD Th2	SD Th2+T	FC Th2/V	FC Th2+T/ Th2	Kruskal-Wallis
**LPC 16:1**	658.521	393.146	591.994	504.143	313.448	153.622	244.057	187.436	0.899	0.852	0.323
**LPC 18:1**	1003.224	638.477	1135.754	756.751	543.617	314.724	419.736	252.621	1.132	0.666	0.264
**LPC 20:4**	228.984	147.839	232.135	194.487	76.333	40.801	77.545	75.187	1.014	0.838	0.151

Variable	V	T	Th2	Th2+T	SD V	SD T	SD Th2	SD Th2+T	FC Th2/V	FC Th2+T/ Th2	Kruskal-Wallis
**SM 32:1;O2**	507.252	606.776	472.607	638.588	213.789	227.562	68.099	246.319	0.932	1.351	0.355
**SM 34:1;O2**	3838.108	4258.493	4101.612	4491.207	1276.181	1525.192	371.994	1410.462	1.069	1.095	0.907
**SM 38:1;O2**	356.317	306.801	321.436	347.154	55.476	57.013	26.778	53.739	0.902	1.080	0.312
**SM 40:2;O2**	168.522	236.503	205.987	243.977	45.734	62.899	35.886	77.555	1.222	1.184	0.114
**SM 40:1;O2**	321.262	330.667	369.799	339.093	44.833	63.442	63.445	87.168	1.151	0.917	0.608
**SM 42:2;O2**	535.778	684.000	500.797	579.418	172.224	206.848	93.556	201.226	0.935	1.157	0.479
**SM 42:1;O2**	476.606	411.638	528.218	488.355	106.666	129.964	105.510	197.125	1.108	0.925	0.397
**SM 44:2;O2**	259.032	288.910	253.861	310.999	79.636	87.126	84.472	136.553	0.980	1.225	0.838
**SM 44:1;O2**	220.649	177.949	240.265	194.015	66.722	54.477	70.120	91.500	1.089	0.808	0.518

Table S8. LPCs and SMs abundance in HEEs treated with tofacitinib and Th2 cytokines and their combination. Mean values and standard deviation (SD) of LPCs and SMs amounts (pmol/mg protein) and p-values obtained after Kruskal-Wallis test (V= vehicle; T= tofacitinib).

Variable	V	T	Th2	Th2+T	SD V	SD T	SD Th2	SD Th2+T	FC Th2/V	FC Th2+T/ Th2	Kruskal-Wallis
**PC 28:0**	72.556	86.635	64.414	86.690	25.869	21.638	2.958	15.349	0.888	1.346	0.121
**PC 30:1**	363.036	414.532	270.569	441.088	111.126	97.212	26.768	62.598	0.745	1.630	**0.025**
**PC 30:0**	429.872	480.167	406.527	493.309	150.149	116.436	45.342	76.664	0.946	1.213	0.242
**PC 32:3**	54.340	69.492	48.518	67.028	23.087	19.537	9.453	15.533	0.893	1.382	0.120
**PC 32:2**	593.076	733.282	403.813	798.889	160.393	175.175	58.762	122.110	0.681	1.978	**0.004**
**PC 32:1**	2279.819	2459.669	2221.289	2646.048	674.509	623.356	187.683	353.756	0.974	1.191	0.375
**PC 32:0**	669.581	692.197	627.625	743.045	172.967	148.260	59.016	77.236	0.937	1.184	0.295
**PC 34:5**	56.899	80.608	39.832	88.712	24.669	31.503	11.638	21.988	0.700	2.227	**0.017**
**PC 34:4**	205.033	264.787	200.554	283.490	101.124	99.151	51.715	63.304	0.978	1.414	0.193
**PC 34:3**	190.221	236.385	195.964	263.818	56.823	59.583	31.520	42.841	1.030	1.346	0.083
**PC 34:2**	3046.460	3357.770	3045.855	3649.991	816.785	804.729	229.870	479.498	1.000	1.198	0.203
**PC 34:1**	3923.240	3909.379	5305.062	4176.788	1020.698	862.900	216.771	469.748	1.352	0.787	**0.005**
**PC 36:6**	37.791	52.499	44.445	55.506	21.403	20.902	21.371	14.353	1.176	1.249	0.365
**PC 36:5**	266.085	353.449	284.163	389.565	130.059	139.674	82.862	96.329	1.068	1.371	0.256
**PC 36:4**	364.474	439.612	538.063	481.705	171.394	157.303	163.863	103.527	1.476	0.895	0.355
**PC 36:3**	341.978	404.727	550.746	466.697	83.106	99.921	141.608	107.146	1.610	0.847	0.058
**PC 36:2**	3798.176	3865.215	5740.706	4151.404	965.132	856.386	299.921	497.056	1.511	0.723	**0.004**
**PC 36:1**	1118.534	974.054	1547.321	1084.716	287.142	184.354	140.640	133.281	1.383	0.701	**0.003**
**PC 38:6**	43.200	58.933	69.472	66.818	16.742	19.063	23.444	13.557	1.608	0.962	0.133
**PC 38:5**	297.971	356.849	500.127	388.550	135.120	124.846	161.422	82.875	1.678	0.777	0.199
**PC 38:4**	108.636	116.699	161.701	127.605	42.696	34.386	46.264	26.909	1.488	0.789	0.207
**PC 38:3**	69.938	75.001	103.151	87.734	15.704	13.277	14.854	10.498	1.475	0.851	**0.010**
**PC 38:2**	512.442	495.778	766.097	540.655	130.654	112.935	78.390	73.817	1.495	0.706	**0.004**
**PC 38:1**	104.608	91.907	134.592	101.430	23.309	16.136	12.891	13.059	1.287	0.754	**0.005**
**PC 40:5**	43.385	49.675	61.669	53.913	17.705	16.876	22.417	11.251	1.421	0.874	0.409
**PC 40:4**	11.331	11.706	15.534	12.921	3.309	3.046	4.386	2.348	1.371	0.832	0.276
**PC 40:2**	59.926	57.750	70.858	62.568	12.587	9.610	8.940	6.624	1.182	0.883	0.203
**PC 40:1**	32.911	24.547	26.969	28.065	6.006	3.373	3.886	4.202	0.819	1.041	0.124

Species	Formula	Number	Number	Molecular Species
**PC 30:1**	C38H74NO8P	30	1	PC(14:0_16:1)
**PC 32:2**	C40H76NO8P	32	2	PC(16:1_16:1)
**PC 34:1**	C42H82NO8P	34	1	PC(16:0_18:1)
**PC 36:1**	C44H86NO8P	36	1	PC(18:0_18:1)
**PC 36:2**	C44H84NO8P	36	2	PC(18:1_18:1)
**PC 38:1**	C46H90NO8P	38	1	PC(18:0_20:1)
**PC 38:2**	C46H88NO8P	38	2	PC(18:1_20:1)
**PC 38:3**	C46H86NO8P	38	3	PC(18:2_20:1)

Table S9. Abundance of PCs members and molecular species characterized for candidate PCs in HEEs treated with tofacitinib and Th2 cytokines and their combination. Mean values and standard deviation (SD) of PCs amounts (pmol/mg protein) and p-values obtained after Kruskal-Wallis test and molecular species characterized for candidate PCs (V= vehicle; T= tofacitinib).

Variable	V	T	Th2	Th2+T	SD V	SD T	SD Th2	SD Th2+T	FC Th2/V	FC Th2+T/ Th2	Kruskal-Wallis
**PE 32:2**	136.331	141.254	48.576	138.623	74.145	78.738	13.255	72.493	0.356	2.854	**0.048**
**PE 32:1**	386.007	365.716	234.543	362.082	177.739	175.920	52.979	168.272	0.608	1.544	0.376
**PE 34:3**	58.063	60.935	37.100	65.857	20.145	21.215	6.588	19.362	0.639	1.775	0.073
**PE 34:2**	1635.223	1709.511	1051.920	1688.017	766.121	857.251	235.204	814.253	0.643	1.605	0.442
**PE 34:1**	1622.136	1572.856	1436.696	1531.582	629.022	677.531	370.660	653.183	0.886	1.066	0.955
**PE 36:4**	37.618	40.812	38.209	44.249	15.762	12.011	13.344	9.335	1.016	1.158	0.821
**PE 36:3**	442.157	496.093	488.639	520.844	116.383	134.168	105.301	100.491	1.105	1.066	0.685
**PE 36:2**	4923.203	5511.188	5826.972	5355.556	1983.609	2593.998	1921.428	2438.002	1.184	0.919	0.848
**PE 36:1**	1177.716	1108.873	1345.298	1143.576	322.841	390.566	407.357	404.933	1.142	0.850	0.790
**PE 38:5**	19.024	19.804	18.182	20.983	16.098	7.666	5.966	6.180	0.956	1.154	0.769
**PE 38:3**	82.499	99.191	93.873	103.175	22.506	29.011	21.060	27.634	1.138	1.099	0.579
**PE 38:2**	483.570	549.925	657.929	561.450	126.688	183.863	225.836	175.020	1.361	0.853	0.584
**PE 40:2**	69.100	84.486	86.385	79.654	13.343	21.622	27.084	21.124	1.250	0.922	0.420
**PE 40:1**	20.373	19.921	21.843	19.713	6.942	5.495	5.865	3.847	1.072	0.902	0.919

Variable	V	T	Th2	Th2+T	SD V	SD T	SD Th2	SD Th2+T	FC Th2/V	FC Th2+T/ Th2	Kruskal-Wallis
**PE O-34:4**	30.288	32.303	26.714	35.276	13.903	10.672	5.534	5.935	0.882	1.321	0.375
**PE O-34:3**	882.260	946.209	731.467	1011.629	345.067	347.086	48.389	259.461	0.829	1.383	0.228
**PE O-34:2**	1608.800	1679.021	1731.419	1779.729	496.662	541.434	222.958	372.096	1.076	1.028	0.932
**PE O-36:5**	55.179	65.169	66.160	72.281	22.172	21.647	14.750	10.768	1.199	1.093	0.475
**PE O-36:4**	293.463	334.247	311.473	375.397	88.333	103.506	60.905	55.429	1.061	1.205	0.279
**PE O-36:3**	1815.346	2012.523	2210.712	2182.253	647.743	699.689	331.716	526.381	1.218	0.987	0.685
**PE O-36:2**	402.089	442.459	492.919	486.941	130.687	132.665	87.795	98.500	1.226	0.988	0.720
**PE O-38:6**	71.383	93.333	97.183	101.250	27.382	29.625	18.969	15.247	1.361	1.042	0.230
**PE O-38:5**	42.491	56.086	59.543	61.133	17.956	16.751	9.671	10.867	1.401	1.027	0.259
**PE O-38:4**	32.836	46.141	41.351	48.162	15.191	15.984	6.990	11.323	1.259	1.165	0.205
**PE O-38:3**	183.085	252.795	261.801	267.557	67.569	78.312	60.071	66.015	1.430	1.022	0.207
**PE O-38:2**	58.137	77.476	67.174	78.248	20.645	21.289	15.818	14.715	1.155	1.165	0.224

Variable	V	T	Th2	T+Th2	SD V	SD T	SD Th2	SD T+Th2	FC Th2/V	FC T+Th2/ Th2	Kruskal-Wallis
**PG 36:1**	28.154	27.109	41.272	28.960	13.660	7.092	14.600	4.796	1.466	0.702	0.215
**PG 36:2**	27.284	24.915	30.874	22.146	15.414	9.088	9.630	8.792	1.132	0.717	0.511
**PG 34:1**	45.986	8.446	58.005	46.309	18.460	5.209	12.912	8.762	1.261	0.798	**0.031**

Variable	V	T	Th2	T+Th2	SD V	SD T	SD Th2	SD T+Th2	FC Th2/V	FC T+Th2/ Th2	Kruskal-Wallis
**PI 36:1**	1335.418	1394.762	1628.428	1335.581	320.494	287.884	214.064	298.802	1.219	0.820	0.186
**PI 32:1**	240.182	240.172	141.570	320.831	154.009	85.501	43.390	134.540	0.589	2.266	0.054
**PI 34:1**	933.958	954.599	778.859	1169.714	317.045	242.225	113.643	247.805	0.834	1.502	0.057

Table S10. Abundance of PEs, PGs and PIs in HEEs treated with tofacitinib and Th2 cytokines and their combination. Mean values and standard deviation (SD) of PEs, PGs, PIs, amounts (pmol/mg protein) and p-values obtained after Kruskal-Wallis test (V= vehicle; T= tofacitinib).

Variable	V	T	Th2	SD V	SD T	SD Th2	SD Th2+T	FC Th2/V	FC Th2+T/ Th2	Kruskal-Wallis
**TG 40:0**	13.748	14.720	10.658	5.951	7.857	2.681	4.751	0.775	1.262	0.613
**TG 42:2**	9.867	12.736	7.131	5.600	8.412	2.010	5.213	0.723	1.575	0.473
**TG 42:1**	57.998	37.264	24.197	43.725	13.574	4.525	7.494	0.417	1.513	**0.040**
**TG 42:0**	18.266	17.486	14.011	5.855	5.440	3.142	3.329	0.767	1.237	0.226
**TG 43:0**	27.150	11.997	11.772	22.872	6.252	6.313	2.940	0.434	1.122	0.468
**TG 44:3**	14.380	16.023	11.722	4.081	1.992	3.915	3.524	0.815	1.467	0.126
**TG 44:2**	35.698	52.843	27.822	12.057	19.053	4.742	11.444	0.779	1.869	**0.008**
**TG 44:1**	60.778	78.539	50.696	12.240	25.529	8.802	7.676	0.834	1.588	**0.010**
**TG 44:0**	38.939	38.488	29.873	15.168	11.546	7.720	13.035	0.767	1.285	0.408
**TG 46:3**	32.585	51.101	21.845	12.772	19.162	3.239	8.540	0.670	2.413	**0.002**
**TG 46:2**	130.229	223.777	97.945	15.600	76.334	16.137	28.377	0.752	2.375	**0.000**
**TG 46:1**	208.646	280.080	157.249	47.533	71.372	18.609	45.746	0.754	1.842	**0.003**
**TG 46:0**	113.681	103.645	77.893	36.478	37.131	21.811	53.199	0.685	1.342	0.321
**TG 47:2**	15.940	17.064	11.844	5.667	4.276	4.666	8.518	0.743	1.549	0.297
**TG 47:1**	39.779	27.559	21.041	18.039	7.127	3.542	8.979	0.529	1.390	**0.034**
**TG 47:0**	36.949	28.546	23.547	14.962	9.276	6.920	9.838	0.637	1.155	0.373
**TG 48:4**	13.035	17.687	9.357	3.815	4.800	1.894	3.157	0.718	2.045	**0.004**
**TG 48:3**	163.642	304.215	93.170	30.202	96.335	11.589	23.688	0.569	3.446	**0.000**
**TG 48:2**	614.596	949.387	396.236	158.423	262.426	41.255	119.700	0.645	2.583	**0.001**
**TG 48:1**	713.438	812.384	468.773	305.980	303.215	71.111	326.580	0.657	1.828	0.110
**TG 48:0**	217.902	182.490	151.770	75.576	81.511	49.412	100.040	0.697	1.293	0.515
**TG 50:5**	8.314	11.277	5.901	2.825	5.152	1.375	5.205	0.710	2.125	0.100
**TG 50:4**	39.244	66.950	27.784	7.395	25.837	6.987	17.431	0.708	2.594	**0.001**
**TG 50:3**	736.315	1245.896	416.695	182.226	368.493	41.428	144.876	0.566	3.327	**0.000**
**TG 50:2**	2052.556	2583.343	1290.322	812.354	831.632	111.755	778.886	0.629	2.153	**0.044**
**TG 50:1**	1158.027	1150.781	821.328	602.823	551.641	234.794	678.343	0.709	1.497	0.613
**TG 52:5**	21.787	33.597	17.051	8.526	16.679	6.932	16.403	0.783	2.299	0.065
**TG 52:4**	114.888	182.596	92.695	17.781	57.145	24.018	39.399	0.807	2.229	**0.001**
**TG 52:3**	1932.404	2768.731	1265.879	637.924	827.802	65.009	561.282	0.655	2.444	**0.002**
**TG 52:2**	2717.757	3077.670	2036.677	1197.180	1167.842	336.002	1250.757	0.749	1.628	0.203
**TG 53:3**	55.032	60.188	38.269	15.756	18.743	5.490	21.315	0.695	1.709	**0.043**
**TG 54:6**	94.428	86.898	87.496	30.510	19.656	9.057	10.106	0.927	1.097	0.539
**TG 54:5**	53.341	59.791	46.122	25.237	26.025	13.419	19.826	0.865	1.446	0.551
**TG 54:4**	169.073	235.225	157.913	24.163	64.167	35.872	25.168	0.934	1.640	**0.006**
**TG 54:3**	2110.295	2724.330	1742.479	746.298	834.777	149.647	666.291	0.826	1.626	**0.031**

Species	Formula	Number	DBs	1	2	3	4	5	6
**TG 44:1**	C47 H88 O6	44	1	TG(12:0_16:0_16:1)	TG(14:0_14:0_16:1)	TG(14:0_16:0_14:1)			
**TG 46:1**	C49 H92 O6	46	1	TG(12:0_16:0_18:1)	TG(14:0_16:0_16:1)	TG(15:0_16:0_15:1)			
**TG 44:2**	C47 H86 O6	44	2	TG(12:0_14:1_18:1)	TG(12:0_16:1_16:1)	TG(14:0_14:1_16:1)	TG(16:0_12:1_16:1)		
**TG 46:2**	C49 H90 O6	46	2	TG(12:0_16:1_18:1)	TG(14:0_14:1_18:1)	TG(14:0_16:1_16:1)	TG(16:0_14:1_16:1)		
**TG 48:2**	C51 H94 O6	48	2	TG(14:0_16:1_18:1)	TG(16:0_16:1_16:1)				
**TG 46:3**	C49 H88 O6	46	3	TG(12:0_16:1_18:2)	TG(14:0_14:1_18:2)	TG(12:1_16:1_18:1)	TG(14:1_14:1_18:1)	TG(14:1_16:1_16:1)	
**TG 48:3**	C51 H92 O6	48	3	TG(14:0_16:1_18:2)	TG(16:0_14:1_18:2)	TG(16:0_16:1_16:2)	TG(14:1_16:1_18:1)	TG(16:1_16:1_16:1)	
**TG 50:3**	C53 H96 O6	50	3	TG(14:0_18:1_18:2)	TG(16:0_14:1_20:2)	TG(16:0_16:1_18:2)	TG(14:1_16:1_20:1)	TG(14:1_18:1_18:1)	TG(16:1_16:1_18:1)
**TG 52:3**	C55 H100 O6	52	3	TG(16:0_18:1_18:2)	TG(20:0_14:1_18:2)	TG(14:1_18:1_20:1)	TG(16:1_16:1_20:1)	TG(16:1_18:1_18:1)	
**TG 50:4**	C53 H94 O6	50	4	TG(14:0_18:2_18:2)	TG(16:0_16:1_18:3)	TG(16:1_16:1_18:2)			
**TG 52:4**	C55 H98 O6	52	4	TG(16:0_18:2_18:2)	TG(16:1_16:1_20:2)	TG(16:1_18:1_18:2)			
**TG 54:4**	C57 H102 O6	54	4	TG(16:1_18:1_20:2)	TG(18:1_18:1_18:2)				

Table S11. TGs amounts and molecular species characterized for candidates TGs in HEEs treated with tofacitinib and Th2 cytokines and their combination. Mean values and standard deviation (SD) of TGs amounts (pmol/mg protein) and p-values obtained after Kruskal-Wallis test and molecular species characterized for candidate TGs. The number of molecular species are reported from 1 to 6 (V= vehicle; T= tofacitinib; DBs = number of double bounds).

Variable	V	T	Th2	Th2+T	SD V	SD T	SD Th2+T	SD Th2	FC Th2/V	FC Th2+T/ Th2	Kruskal-Wallis
**Cholesterol Sulfate**	3650.722	3476.127	4601.769	3794.5	648.154	547.234	315.653	1029.698	1.261	0.825	0.227

Variable	V	T	Th2	Th2+T	SD V	SD T	SD Th2	SD Th2+T	FC Th2/V	FC Th2+T/ Th2	Kruskal-Wallis
**Cer[NS]34:2**	73.093	75.599	73.267	74.492	47.115	45.175	43.464	44.82	1.002	1.017	0.998
**Cer[NS]35:2**	3.595	2.834	3.404	2.867	3.851	2.87	3.846	3.274	0.947	0.842	0.717
**Cer[NS]36:2**	99.992	106.255	92.375	104.254	45.205	37.735	34.44	26.119	0.924	1.129	0.883
**Cer[NS]37:2**	2.423	2.049	1.583	2.171	0.684	0.684	0.535	0.995	0.653	1.371	0.344
**Cer[NS]38:2**	68.854	65.654	73.585	61.931	49.354	42.75	61.538	39.339	1.069	0.842	1
**Cer[NS]39:2**	4.571	5.531	4.389	4.622	2.234	2.275	2.289	2.932	0.96	1.053	0.79
**Cer[NS]40:2**	207.305	225.463	246.077	214.712	151.579	150.379	212.334	142.932	1.187	0.873	0.917
**Cer[NS]41:2**	18.435	20.728	16.293	21.057	9.645	9.521	9.777	10.543	0.884	1.292	0.895
**Cer[NS]42:2**	895.153	844.043	904.436	809.028	675.165	568.659	661.799	273.798	1.01	0.895	0.995
**Cer[NS]43:2**	22.685	24.437	19.503	25.367	10.145	7.206	9.989	5.868	0.86	1.301	0.829
**Cer[NS]44:2**	611.793	629.392	615.463	563.926	591.665	564.666	503.855	198.622	1.006	0.916	0.792
**Cer[NS]45:2**	15.161	14.94	9.822	13.73	9.286	7.815	5.442	6.883	0.648	1.398	0.767
**Cer[NS]46:2**	632.81	564.18	382.411	410.095	504.994	387.958	286.661	173.072	0.604	1.072	0.79
**Cer[NS]47:2**	8.362	9.059	4.969	9.334	5.311	4.501	2.479	5.624	0.594	1.878	0.279
**Cer[NS]48:2**	415.6	413.619	269.841	328.667	334.625	263.752	177.822	172.428	0.649	1.218	0.721
**Cer[NS]49:2**	4.173	3.702	1.699	3.964	2.474	2.25	1.149	1.863	0.407	2.333	0.085
**Cer[NS]50:2**	79.378	96.657	61.049	77.936	64.058	59.918	34.806	43.762	0.769	1.277	0.728
**Cer[NS]32:1**	301.571	306.68	261.204	259.79	155.768	138.947	134.713	68.487	0.866	0.995	0.876
**Cer[NS]33:1**	37.155	36.827	29.52	35.613	14.027	8.384	8.135	6.03	0.794	1.206	0.373
**Cer[NS]34:1**	3249.15	3637.743	3146.112	3328.847	1258.018	1228.447	1362.937	552.05	0.968	1.058	0.944
**Cer[NS]35:1**	9.795	8.342	6.957	9.874	3.805	3.904	2.684	0.831	0.71	1.419	0.239
**Cer[NS]36:1**	750.66	791.208	661.577	814.007	338.093	246.068	238.329	227.072	0.881	1.23	0.825
**Cer[NS]37:1**	15.895	14.602	9.708	15.518	8.664	4.203	2.268	3.803	0.611	1.598	0.085
**Cer[NS]38:1**	846.817	915.861	708.212	973.155	354.816	246.001	186.178	210.44	0.836	1.374	0.254
**Cer[NS]39:1**	50.25	51.227	34.081	59.099	28.625	25.536	14.371	31.733	0.678	1.734	0.532
**Cer[NS]40:1**	2728.849	2771.405	2539.89	2878.591	1394.637	1029.195	1309.535	1156.533	0.931	1.133	0.819
**Cer[NS]41:1**	202.397	198.566	136.578	223.632	115.138	94.559	60.074	112.471	0.675	1.637	0.401
**Cer[NS]42:1**	6327.418	6287.925	5911.758	6218.356	3423.084	2432.674	3252.343	2632.491	0.934	1.052	0.96
**Cer[NS]43:1**	274.014	274.445	205.207	305.321	154.335	128.047	93.442	165.914	0.749	1.488	0.539
**Cer[NS]44:1**	5680.251	5547.25	5545.631	5420.329	2875.593	2167.24	3053.903	2285.251	0.976	0.977	0.913
**Cer[NS]45:1**	70.324	70.27	54.275	74.358	41.807	42.14	28.936	44.005	0.772	1.37	0.838
**Cer[NS]46:1**	987.304	954.324	924.676	1060.45	635.873	536.193	599.545	680.586	0.937	1.147	0.79
**Cer[NS]47:1**	15.377	11.912	9.638	15.697	8.726	9.197	6.075	9.822	0.627	1.629	0.377
**Cer[NS]48:1**	168.054	159.653	145.576	173.85	111.824	106.528	98.647	110.876	0.866	1.194	0.846
**Cer[NS]49:1**	4.175	3.134	2.32	4.61	1.623	1.658	1.012	1.444	0.556	1.987	0.066
**Cer[NS]50:1**	33.223	35.345	29.776	32.416	20.771	15.79	14.205	13.306	0.896	1.089	0.958

Variable	V	T	Th2	Th2+T	SD V	SD T	SD Th2	SD Th2+T	FC Th2/V	FC Th2+T/ Th2	Kruskal-Wallis
**Cer[NDS]32:0**	22.243	17.731	16.158	17.403	5.769	3.725	6.043	2.816	0.726	1.077	0.156
**Cer[NDS]33:0**	1.775	1.255	1.052	1.331	1.657	0.654	0.767	0.968	0.593	1.265	0.743
**Cer[NDS]34:0**	124.528	111.921	104.56	126.486	49.94	31.246	25.605	5.251	0.84	1.21	0.502
**Cer[NDS]35:0**	1.006	0.662	0.732	0.705	0.189	0.325	0.35	0.345	0.728	0.962	0.33
**Cer[NDS]36:0**	50.13	47.064	46.391	52.07	34.645	29.87	30.598	30.85	0.925	1.122	0.748
**Cer[NDS]37:0**	2.244	2.213	1.946	2.931	1.58	1.296	1.836	2.137	0.867	1.506	0.843
**Cer[NDS]38:0**	62.953	58.576	57.03	68.462	46.625	37.896	44.826	53.553	0.906	1.2	0.909
**Cer[NDS]39:0**	4.547	3.238	2.719	3.856	2.186	1.641	1.034	1.57	0.598	1.418	0.447
**Cer[NDS]40:0**	156.69	140.152	146.606	183.942	99.017	90.78	122.132	98.545	0.936	1.255	0.819
**Cer[NDS]41:0**	8.809	6.723	6.909	9.685	4.481	3.935	4.768	4.492	0.784	1.402	0.414
**Cer[NDS]42:0**	185.888	176.797	215.4	238.121	101.903	78.386	134.566	107.864	1.159	1.105	0.533
**Cer[NDS]43:0**	7.053	6.486	6.246	7.093	3.592	3.626	4.634	4.044	0.886	1.136	0.732
**Cer[NDS]44:0**	171.079	140.342	166.334	161.279	114.903	103.032	145.534	110.995	0.972	0.97	0.96
**Cer[NDS]45:0**	7.241	5.159	4.639	5.603	3.771	2.259	2.03	2.438	0.641	1.208	0.416
**Cer[NDS]46:0**	84.237	73.053	78.842	87.398	51.939	48.778	65.033	56.567	0.936	1.109	0.932
**Cer[NDS]47:0**	6.057	4.813	3.695	4.264	3.5	1.232	1.006	1.327	0.61	1.154	0.384
**Cer[NDS]48:0**	50.387	43.985	41.89	48.108	30.351	27.409	30.155	30.918	0.831	1.148	0.845
**Cer[NP]38:0**	120.277	121.364	138.344	129.861	48.914	30.418	38.207	15.734	1.15	0.939	0.74
**Cer[NP]39:0**	8.844	9.847	8.074	9.693	4.799	3.514	2.752	0.985	0.913	1.2	0.515
**Cer[NP]40:0**	325.907	313.89	373.332	335.014	127.111	86.506	183.495	84.635	1.146	0.897	0.983
**Cer[NP]41:0**	30.418	30.305	25.208	30.271	13.378	11.285	7.288	6.249	0.829	1.201	0.69
**Cer[NP]42:0**	1091.252	963.1	1173.769	1043.273	308.396	158.583	402.161	147.719	1.076	0.889	0.742
**Cer[NP]43:0**	28.291	26.486	24.5	27.327	10.915	7.625	8.857	6.963	0.866	1.115	0.889
**Cer[NP]44:0**	509.72	493.832	550.248	468.123	118.994	115.707	163.703	70.469	1.08	0.851	0.739
**Cer[NP]45:0**	12.053	10.076	10.602	9.251	6.11	2.393	2.004	1.701	0.88	0.873	0.771
**Cer[NP]46:0**	397.667	375.841	376.959	305.995	403.732	469.971	426.197	201.981	0.948	0.812	0.819
**Cer[NP]47:0**	10.4	7.205	6.993	7.472	5.398	1.66	0.971	0.901	0.672	1.068	0.588
**Cer[NP]48:0**	333.553	375.258	249.865	275.607	218.338	223.088	237.442	76.971	0.749	1.103	0.44
**Cer[NP]49:0**	4.322	3.013	2.314	3.051	2.651	0.63	0.837	0.576	0.535	1.319	0.261
**Cer[NP]50:0**	71.53	94.931	60.187	80.682	23.897	29.314	38.027	11.615	0.841	1.341	0.314
**Cer[AH]34:1**	9.668	10.907	10.274	9.21	8.322	9.523	10.136	6.268	1.063	0.896	0.933
**Cer[AH]36:1**	31.283	36.848	31.93	32.426	24.476	30.66	29.343	22.261	1.021	1.016	0.974
**Cer[AH]38:1**	6.654	5.795	5.309	5.428	4.185	3.578	3.633	3.127	0.798	1.022	0.668
**Cer[AH]40:1**	27.262	28.046	15.509	34.682	30.837	34.181	14.128	41.542	0.569	2.236	0.688
**Cer[AH]42:1**	46.395	43.029	24.396	48.608	48.844	46.814	19.236	55.938	0.526	1.992	0.854
**Cer[AH]44:1**	33.212	31.931	23.919	31.122	13.512	12.948	5.921	14.039	0.72	1.301	0.659
**Cer[AH]46:1**	14.543	13.463	11.96	11.497	9.912	8.448	7.947	6.638	0.822	0.961	0.766
**Cer[AP]38:0**	4.9	3.965	3.745	4.198	2.645	1.224	1.397	0.608	0.764	1.121	0.69
**Cer[AP]40:0**	8.086	8.212	7.295	7.824	4.796	2.779	3.043	1.073	0.902	1.073	0.809
**Cer[AP]42:0**	30.3	26.08	25.674	24.297	14.067	4.768	6.01	4.218	0.847	0.946	0.901
**Cer[AP]44:0**	15.671	12.023	12.558	11.972	7.511	2.272	2.628	1.115	0.801	0.953	0.883
**Cer[AP]46:0**	6.465	4.368	3.902	3.493	4.837	0.613	0.949	0.707	0.603	0.895	0.178

Variable	V	T	Th2	Th2+T	SD V	SD T	SD Th2	SD Th2+T	FC Th2/V	FC Th2+T/ Th2	Kruskal-Wallis
**Cer[NH]40:1**	15.781	22.945	18.614	20.763	9.894	13.223	16.346	8.931	1.18	1.115	0.435
**Cer[NH]41:1**	3.905	4.458	3.362	4.072	1.481	1.93	2.426	1.947	0.861	1.211	0.653
**Cer[NH]42:1**	119.129	117.868	102.385	99.059	57.916	50.129	45.253	35.138	0.859	0.968	0.655
**Cer[NH]43:1**	7.172	7.237	4.961	5.578	2.698	1.628	2.619	2.67	0.692	1.125	0.267
**Cer[NH]44:1**	110.61	108.108	96.841	88.834	62.952	54.335	47.889	21.773	0.876	0.917	0.94
**Cer[NH]45:1**	3.441	3.262	2.112	3.193	0.718	0.908	0.799	0.713	0.614	1.512	**0.044**
**Cer[NH]46:1**	81.387	83.714	72.001	62.905	34.765	34.486	34.94	14.608	0.885	0.874	0.755
**Cer[AS]34:1**	117.714	132.066	114.854	161.501	39.469	31.909	19.386	25.331	0.976	1.406	0.069
**Cer[AS]35:1**	23.264	22.506	21.387	21.957	17.34	17.922	16.496	16.362	0.919	1.027	0.864
**Cer[AS]36:1**	10.205	13.639	7.429	14.634	6.763	7.804	3.734	5.518	0.728	1.97	0.167
**Cer[AS]37:1**	3.52	4.243	1.822	4.045	2.141	1.693	2.243	1.198	0.518	2.22	0.191
**Cer[AS]38:1**	60.886	65.247	38.235	74.814	52.906	30.246	21.46	20.839	0.628	1.957	0.097
**Cer[AS]39:1**	20.016	22.022	11.629	25.183	16.328	11.058	10.583	5.084	0.581	2.166	0.252
**Cer[AS]40:1**	199.794	206.556	127.798	235.23	139.45	82.476	49.141	79.342	0.64	1.841	0.128
**Cer[AS]41:1**	42.887	52.305	21.646	59.228	33.898	22.787	19.325	20.537	0.505	2.736	0.107
**Cer[AS]42:1**	350.2	369.315	214.81	406.995	230.083	120.963	78.674	117.35	0.613	1.895	0.077
**Cer[AS]43:1**	32.142	35.475	16.36	38.451	23.173	14.089	12.784	13.279	0.509	2.35	0.228
**Cer[AS]44:1**	203.249	210.641	156.594	217.456	109.535	62.928	51.616	77.141	0.77	1.389	0.449
**Cer[AS]45:1**	8.323	8.86	4.269	8.17	4.035	3.02	2.546	2.253	0.513	1.914	0.088
**Cer[AS]46:1**	43.456	40.239	34.612	43.903	28.578	24.347	23.09	28.069	0.797	1.268	0.87

Variable	V	T	Th2	Th2+T	SD V	SD T	SD Th2	SD Th2+T	FC Th2/V	FC Th2+T/ Th2	Kruskal-Wallis
**Cer[EODS]64:2**	31.302	29.801	28.152	25.798	45.79	43.678	42.006	38.945	0.899	0.916	0.872
**Cer[EODS]66:2**	158.978	153.396	112.118	114.578	134.485	89.724	62.497	68.331	0.705	1.022	0.779
**Cer[EODS]68:2**	107.131	115.202	83.175	90.78	54.337	47.603	42.881	18.673	0.776	1.091	0.695
**Cer[EOS]64:3**	222.846	204.009	156.529	161.03	191.448	140.365	107.059	101.839	0.702	1.029	0.809
**Cer[EOS]66:3**	965.313	897.068	679.523	649.214	839.596	664.375	482.775	390.776	0.704	0.955	0.782
**Cer[EOS]68:3**	266.899	338.615	273.418	269.888	225.079	325.325	275.482	191.121	1.024	0.987	0.867
**Cer[EOH]64:3**	19.102	21.791	10.717	21.014	7.773	5.688	6.161	7.971	0.561	1.961	0.068
**Cer[EOH]66:3**	59.624	75.666	39.248	71.981	31.076	16.558	22.617	24.608	0.658	1.834	0.121
**Cer[EOH]68:3**	72.078	83.497	44.781	84.792	42.24	18.687	20.096	23.455	0.621	1.893	0.093
**Cer[EOP]64:2**	18.306	17.827	12.996	16.097	7.642	4.855	4.765	3.484	0.71	1.239	0.351
**Cer[EOP]66:2**	75.438	74.92	51.777	62.004	31.645	35.604	38.666	11.123	0.686	1.198	0.218
**Cer[EOP]68:2**	82.664	84.365	59.029	82.729	28.823	31.682	26.61	14.444	0.714	1.401	0.355

Variable	V	T	Th2	Th2+T	SD V	SD T	SD Th2	SD Th2+T	FC Th2/V	FC Th2+T/ Th2	Kruskal-Wallis
**CerNS(16)**	14.462	13.722	14.104	14.5	1.766	2.164	2.487	1.007	0.975	1.028	0.454
**CerNS(17)**	0.892	0.842	0.835	0.849	0.191	0.131	0.153	0.124	0.936	1.017	0.97
**CerNS(18)**	70.48	65.164	65.854	68.558	7.013	5.977	7.085	5.228	0.934	1.041	0.256
**CerNS(19)**	0.15	0.128	0.139	0.148	0.03	0.011	0.018	0.015	0.928	1.061	0.22
**CerNS(20)**	3.172	2.891	3.097	3.23	1.002	0.662	0.748	0.729	0.976	1.043	0.795
**CerNS(22)**	1.108	1.031	1.092	1.048	0.698	0.325	0.532	0.24	0.986	0.96	0.95
**CerNS(24)**	0.212	0.137	0.156	0.156	0.112	0.07	0.086	0.046	0.737	0.995	0.449
**CerNP(16)**	2.469	2.286	2.433	2.353	0.267	0.327	0.428	0.324	0.986	0.967	0.804
**CerNP(18)**	13.963	12.388	12.604	12.776	1.147	1.041	1.512	1.196	0.903	1.014	0.064
**CerNP(20)**	2.391	1.864	2.364	2.036	0.699	0.335	0.553	0.628	0.989	0.862	0.259

Table S12. Cholesterol sulfate, ceramides and of the selected SBs amounts in HEEs treated with tofacitinib and Th2 cytokines and their combination. Mean values and standard deviation (SD) of Cholesterol Sulfate, ceramides and of the selected SBs amounts (pmol/mg protein) and p-values obtained after Kruskal-Wallis test (V= vehicle; T= tofacitinib).
